# Binding of hairpin pyrrole and imidazole polyamides to DNA: relationship between torsion angle and association rate constants

**DOI:** 10.1093/nar/gks897

**Published:** 2012-10-05

**Authors:** Yong-Woon Han, Tomoko Matsumoto, Hiroaki Yokota, Gengo Kashiwazaki, Hironobu Morinaga, Kaori Hashiya, Toshikazu Bando, Yoshie Harada, Hiroshi Sugiyama

**Affiliations:** ^1^Institute for Integrated Cell-Material Sciences (WPI-iCeMS), Kyoto University, Yoshida Honmachi, Sakyo, Kyoto 606-8501, ^2^Department of Human Life Studies, Doshisha Women’s College of Liberal of Arts, Teramachi Nishiiru, Imadegawa-dori, Kamigyo, Kyoto 602-0893, ^3^Department of Chemistry, Graduate School of Science, Kyoto University, Kitashirakawa-oiwakecho, Sakyo, Kyoto 606-8502 and ^4^CREST, Japan Science and Technology Corporation (JST), Sanbancho, Chiyoda, Tokyo 102-0075, Japan

## Abstract

*N*-methylpyrrole (Py)-*N*-methylimidazole (Im) polyamides are small organic molecules that bind to DNA with sequence specificity and can be used as synthetic DNA-binding ligands. In this study, five hairpin eight-ring Py–Im polyamides **1–5** with different number of Im rings were synthesized, and their binding behaviour was investigated with surface plasmon resonance assay. It was found that association rate (*k*_a_) of the Py–Im polyamides with their target DNA decreased with the number of Im in the Py–Im polyamides. The structures of four-ring Py–Im polyamides derived from density functional theory revealed that the dihedral angle of the Py amide carbonyl is 14∼18°, whereas that of the Im is significantly smaller. As the minor groove of DNA has a helical structure, planar Py–Im polyamides need to change their conformation to fit it upon binding to the minor groove. The data explain that an increase in planarity of Py–Im polyamide induced by the incorporation of Im reduces the association rate of Py–Im polyamides. This fundamental knowledge of the binding of Py–Im polyamides to DNA will facilitate the design of hairpin Py–Im polyamides as synthetic DNA-binding modules.

## INTRODUCTION

*N*-Methylpyrrole (Py)-*N*-methylimidazole (Im) polyamides are small organic molecules that can recognize specific DNA sequences in the minor groove of B-form DNA, according to DNA recognition rules (1,2). Py favours T, A and C bases, whereas Im favours a G base. A lone electron pair on N-3 in Im forms a hydrogen bond with the 2-amino hydrogen of guanine (G). Thus, antiparallel pairings of Im/Py and Py/Im specify G•C and C•G, respectively, and antiparallel pairings of Py/Py specify A•T or T•A degenerately (1,2). Aliphatic β-alanine (β) can be substituted for Py. It has been used effectively when molecules have more than five consecutive Py or Im residues, by adjusting the pitch between amide bonds of Py–Im polyamides and the accepting residue of the minor groove. Antiparallel pairings of Py/β and β/Py specify A•T or T•A degenerately, and antiparallel pairings of Im/β and β/Im specify G•C and C•G, respectively (3,4).

As Py–Im polyamides can bind to DNA with sequence specificity comparable with DNA-binding proteins, they can be substituted for the DNA-binding domain of a transcription factor. Py–Im polyamides that can bind to a promoter region have been designed to inhibit gene expression (5–9). Furthermore, Py–Im polyamides have been conjugated with a peptide or a small organic molecule to create synthetic transcriptional activators that stimulate gene expression (10–13). The dissociation equilibrium constants (*K*_D_s) of these Py–Im polyamides with their target DNA sequences were extensively determined, by DNase I footprinting, by the Dervan group. However, only a few of their corresponding association rate constants and dissociation rate constants have been reported (14–16). It may be crucial for the design of a synthetic DNA-binding module to determine not only *K*_D_s but also the association rate constant (*k*_a_) and the dissociation rate constant (*k*_d_) of Py–Im polyamides because the association/dissociation rate constants are contingent on respective transcription factors (17,18).

A zinc finger is a representative transcription factor DNA-binding domain that binds to GC-rich sequences (19). Many eukaryotic genes contain highly GC-rich sequences in the promoter region (20); thus, it is potentially valuable to synthesize Py–Im polyamides that recognize the GC-rich promoter region. However, 5′-GCGC-3′ and 5′-CGCG-3′ have been identified as difficult recognition sequences of Py–Im polyamides (3,21). It is not known why these sequences are difficult to recognize.

In this study, we synthesized five 8-ring Py–Im polyamides **1–5** with different numbers of Im rings, and we measured the *k*_a_ and *k*_d_ values of the DNA-Py–Im polyamide complexes by surface plasmon resonance (SPR) assay. We also estimated the structures of four-ring Py–Im polyamides by density functional theory (DFT) and *ab initio* quantum chemical calculation. Our SPR data and the calculated structures elucidated the relationship between the structure of the Py–Im polyamides and the association rate of the Py–Im polyamides with their target DNA.

## MATERIALS AND METHODS

### General methods

The following abbreviations apply: Fmoc, fluorenylmethoxycarbonyl; DMSO, dimethyl sulphoxide; TFA, trifluoroacetic acid; β, β-alanine; γ, γ-aminobutyric acid; Py, *N*-methylpyrrole; Im, *N*-methylimidazole; Dp, *N*, *N*-dimethyl-1,3-propylamine.

Electrospray ionization time-of-flight mass spectrometry (ESI-TOFMS) was carried out on a BioTOF II (Bruker Daltonics) mass spectrometer to determine the molecular weight of Py–Im polyamides **1–5**.

### Polyamide synthesis

Py–Im polyamides **1–5** were synthesized in a stepwise reaction using a previously described Fmoc solid-phase protocol (21). Syntheses were performed using a pioneer peptide synthesizer (PSSM-8, Shimadzu) with a computer-assisted operation system on a 36 µM scale (100 mg of Fmoc-β-alanine Wang resin). After the synthesis, Dp was mixed with the resin for 4 h at 55°C and the mixture was shaken at 550 r.p.m. to detach the Py-–Im polyamides from the resin. Purification of Py-–Im polyamides **1–5** was performed using a high-performance liquid chromatography (HPLC) PU-2080 Plus series system (JASCO), using a 10 × 150 mm ChemcoPak Chemcobond 5-ODS-H reverse-phase column in 0.1% TFA in water, with acetonitrile as eluent, at a flow rate of 3 ml/min and a linear gradient elution of 20–60% acetonitrile >20 min, with detection at 254 nm. Collected fractions were analysed by ESI-TOFMS.

### SPR assay

All SPR experiments were performed on a BIACORE X instrument at 25°C as described previously (21,22). The sequences of biotinylated hairpin DNAs containing target sequences are shown in Figure 2 and Supplementary Figure S1. The hairpin DNAs were immobilized on a streptavidin-coated SA sensor chip at a flow rate of 20 µl/min to obtain the required immobilization level (up to ∼1400 resonance units (RU) rise). Experiments were carried out using HBS-EP (10 mM 4-(2-hydroxyethyl)-1-piperazineethanesulfonic acid (HEPES), 150 mM NaCl, 3 mM ethylenediaminetetraacetic acid and 0.005% Surfactant P20) buffer with 0.1% DMSO at 25°C, pH 7.4. A series of sample solutions were prepared in HBS-EP buffer with 0.1% DMSO and were injected at a flow rate of 20 µl/min. To measure association and dissociation rate constants (*K*_D_, *k*_a_ and *k*_d_), data processing was performed with an appropriate fitting model using the BIAevaluation 4.1 program. The sensorgrams of all data were fitted by using the 1:1 binding model with mass transfer. The values of *K*_D_, *k*_a_ and *k*_d_ for all data are summarized in Table 1 and Supplementary Table S1.

**Table 1.** Binding affinity of **1–5**

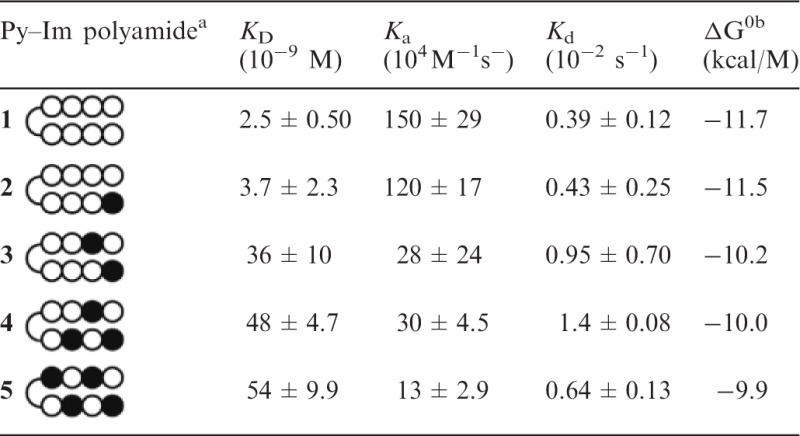

^a^Closed circle and open circle indicate Im and Py, respectively.

^b^ΔG^0^ values are calculated from the equation, ΔG^0 ^= −RTln (1/*K*_D_), where R is universal gas constant, 1.987 cal/M•K; T is absolute temperature in a unit of K, here 298.15 K.

### Structural model calculation

DFT and Hartree–Fock calculations were performed with the Gaussian 09 software package. Structural energy was first minimized by means of a Parameterized Model number 3 (PM3) model, followed by DFT and a 6-311+G* polarization basis set or the Hartree–Fock method and a 6-31G* polarization basis set.

## RESULTS AND DISCUSSION

### Hairpin eight-ring Py–Im polyamide synthesis

**Figure 1.**
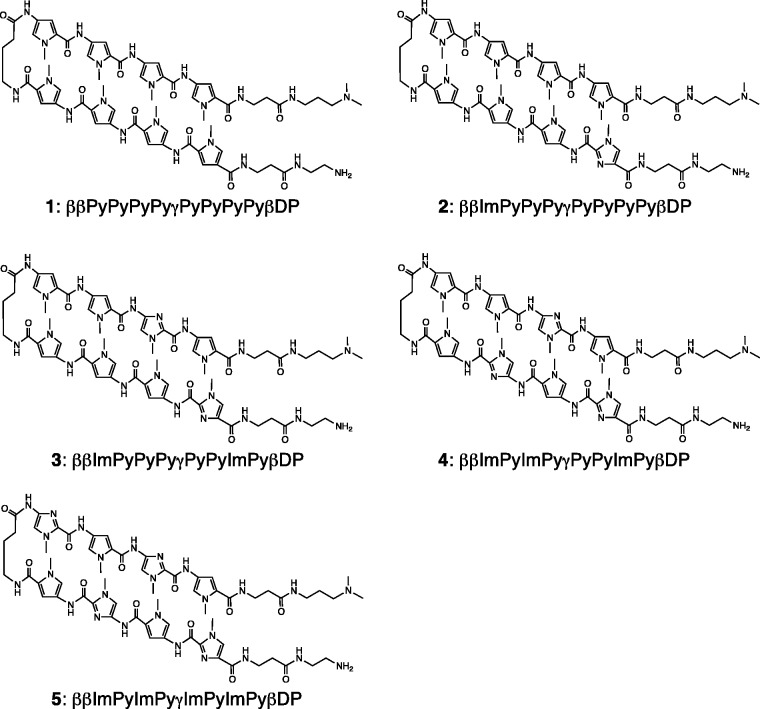
Structures of Py–Im polyamides **1–5**.

**Figure 2.**
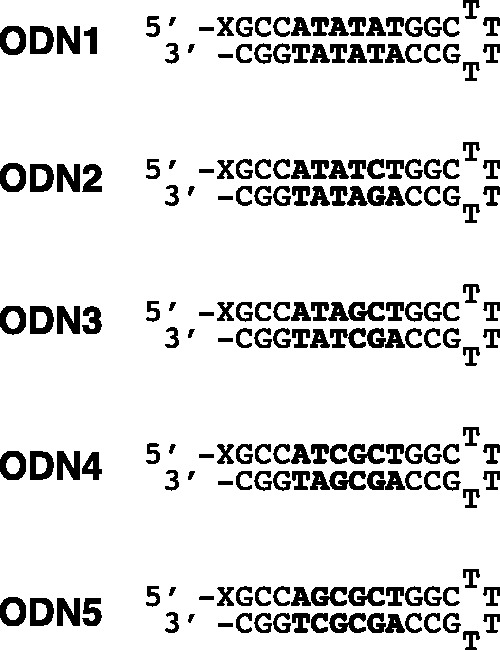
Sequences of 5′-biotinylated hairpin DNAs (ODN1–5). X represents biotin. The binding sequences of the Py–Im polyamides are shown in bold.

To investigate the binding properties of Py and Im in hairpin Py–Im polyamides, we designed and synthesized five hairpin eight-ring Py–Im polyamides **1–5** (Figure 1) by the Fmoc-chemistry solid-phase synthesis method. Two β-alanines were attached to the N-terminal of **1–5** (Figure 1) for the optional construction of a fluorescence Py–Im polyamide conjugate (Han *et al.*, unpublished data). We purified **1–5** by reverse-phase HPLC, and then confirmed that the purity of **1–5** was >95% by analytical HPLC and ESI-TOFMS. The β-Dp linker at the C-terminal has ∼100-fold steric preference for A•T or T•A relative to G•C or C•G (23). Based on the recognition rule of polyamides, the target DNA sequences of **1–5** are 5′-WWWWWW-3′, 5′-WWWWCW-3′, 5′-WWWGCW-3′, 5′-WWCGCW-3′ and 5′-WGCGCW-3′, respectively, and we prepared five 5′-biotinylated hairpin DNAs (ODN1–5) (Figure 2). However, because of two β-alanines attached to the N-terminal of **1–5**, steric hinderance between the tails of **1–5** and the DNA minor groove may suppress the steric preference for A•T or T•A relative to G•C or C•G. To characterize the effect of the two β-alanines, we also prepared five 5′-biotinylated hairpin DNAs (ODN6–10) (Supplementary Figure S1).

### SPR assay

**Figure 3.**
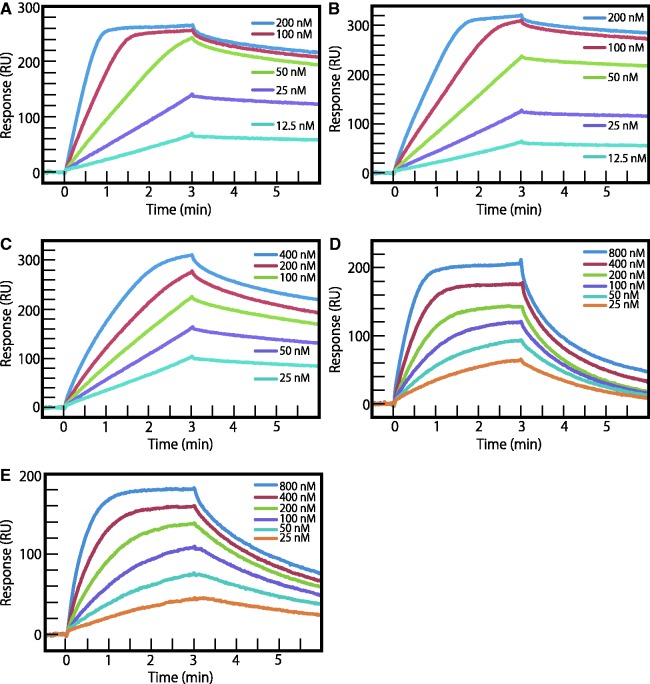
SPR sensorgrams for the interaction of Py–Im polyamides with hairpin DNAs immobilized on a sensor chip SA. (**A**) Py–Im polyamide **1** with ODN1 at a concentration range from 1.25 × 10^−8^ (lowest curve) to 2.0 × 10^−7^ M (highest curve). (**B**) Py–Im polyamide **2** with ODN2 at a concentration range from 1.25 × 10^−8^ (lowest curve) to 2.0 × 10^−7^ M (highest curve). (**C**) Py–Im polyamide **3** with ODN3 at a concentration range from 2.5 × 10^−8^ (lowest curve) to 4.0 × 10^−7^ M (highest curve). (**D**) Py–Im polyamide **4** with ODN4 at a concentration range from 2.5 × 10^−8^ (lowest curve) to 8.0 × 10^−7^ M (highest curve). (**E**) Py–Im polyamide **5** with ODN5 at a concentration range from 2.5 × 10^−8^ (lowest curve) to 8.0 × 10^−7^ M (highest curve).

To measure *K*_D_, *k*_a_ and *k*_d_ values of **1–5** for their target DNA, we performed SPR assays as described in the ‘Materials and Methods’ section. The five 5′-biotinylated hairpin DNAs (ODN1–5) (Figure 2) that include the target DNA sequences were immobilized on a streptavidin-coated sensor chip, and the Py–Im polyamide solutions were injected. As shown in Figure 3, SPR sensorgrams were obtained, and the kinetic binding parameters *K*_D_, *k*_a_ and *k*_d_ were determined (Table 1). The *K*_D_s of **1–5** increased with an increase in the number of Im as follows: 2.5 × 10^−^^9^, 3.7 × 10^−^^9^, 3.6 × 10^−^^8^, 4.8 × 10^−^^8^ and 5.4 × 10^−^^8^ M, respectively. Interestingly, the binding affinities of **1–4** were 22, 15, 1.5 and 1.1-fold, respectively, over that of **5**. Of importance was that the *k*_d _values of **1–5** were comparable with each other (0.0039∼0.014 s^−^^1^) (Table 1), whereas the *k*_a_ values of **1–5** were 1.5 × 10^6^, 1.2 × 10^6^, 2.8 × 10^5^, 3.0 × 10^5^ and 1.3 × 10^5 ^M^−^^1^s^−^^1^, respectively (Table 1). These results indicate that the association rate of the Py–Im polyamides with their target DNA decreased as the number of Im in the Py–Im polyamides increased. However, once **1–5** bound to their target DNAs, the dissociation rates of the Py–Im polyamides from the respective complexes were comparable with each other. We also determined the free energy change (ΔG°, kcal/M) from the *K*_D_ on the formation of the Py–Im polyamides **1–5**/DNA complexes (Table 1). The ΔG° values of **1**/ODN1, **2**/ODN2, **3**/ODN3, **4**/ODN4 and **5**/ODN5 were −11.7, −11.5, −10.2, −10.0 and −9.9 kcal/M, respectively.

We also prepared five 5′-biotinylated hairpin DNAs (ODN6–10) (Supplementary Figure S1) to characterize the effect of the two β-alanines, and as shown in Supplementary Figure S2 and Supplementary Table S1, we determined the kinetic binding parameters *K*_D_, *k*_a_ and *k*_d_. As described earlier in the text, the *K*_D_s of **1–5** also increased with an increase in the number of Im as follows: 6.1 × 10^−^^9^, 1.3 × 10^−^^8^, 2.5 × 10^−^^8^, 1.1 × 10^−^^7^ and 2.5 × 10^−^^7^ M, respectively, and the binding affinities of **1–4** were 41, 19, 10 and 2.3-fold, respectively, over that of **5**. The binding affinities of **1–5** to ODN6-10 were reduced 2.3, 3.5, 0.72, 2.3 and 4.6-fold, respectively, compared with those to ODN1-5 (Table 1 and Supplementary Table S1), indicating that the β-Dp linker at the C-terminal, with two β-alanines attached to the N-terminal, has a slight steric preference for A•T or T•A relative to G•C or C•G. The *k*_d _values of **1–5** were also comparable with each other (0.011∼0.023 s^−^^1^) (Supplementary Table S1), whereas the *k*_a_ values of **1–5** were 1.8 × 10^6^, 1.8 × 10^6^, 5.1 × 10^5^, 1.8 × 10^5^ and 8.9 × 10^4 ^M^−^^1^s^−^^1^, respectively (Supplementary Table S1). The ΔG° values of **1**/ODN6, **2**/ODN7, **3**/ODN8, **4**/ODN9 and **5**/ODN10 were −11.2, −10.8, −10.4, −9.5 and −9.0 kcal/M, respectively.

Previously, Crothers and coworkers reported the *k*_a_ and *k*_d_ values of ImPyPyPy-γ-ImPyPyPy-β-Dp, and the *k*_d_ value was 0.002 s^−^^1^, which is consistent with our data. However, the *k*_a_ value was 7.0 × 10^7 ^M^−^^1^s^−^^1^ and 46-fold times higher than that of **1**. Dervan and coworkers (24) suggested that the Im located at the C-terminal end of each four-ring Py–Im polyamide subunit is somehow less capable of strong hydrogen bond formation than the N-terminal residues. Therefore, the binding affinity of ImPyPyPy-γ-ImPyPyPy-β-Dp may be relatively high, like that of ImImPyPy-γ-ImImPyPy-β-Dp as discussed later in the text.

### Structures of four-ring Py–Im polyamide estimated by density functional theory

**Figure 4.**
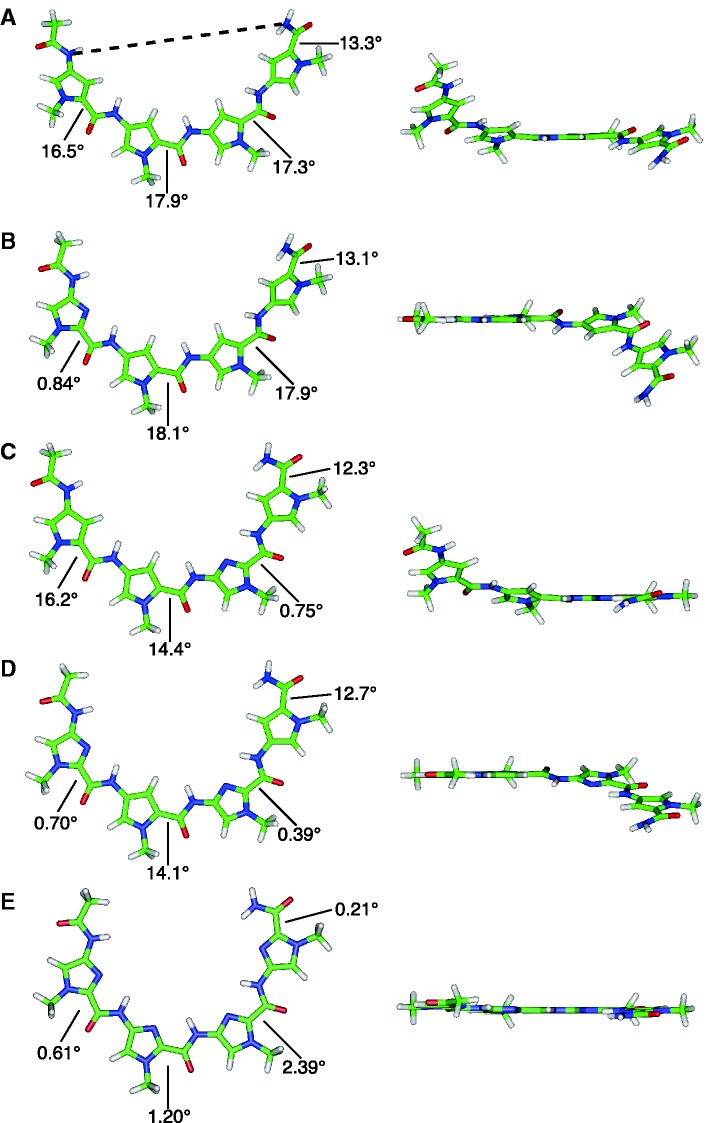
Models of four-ring polyamide subunits calculated by density functional theory. Angles indicate the dihedral angles between each Py or Im and the contiguous amide (N1-C2-CO-O). (**A**) Py-Py-Py-Py. Dotted line indicates the N-to-N distance at both ends of the four-ring polyamide. (**B**) Im-Py-Py-Py, (**C**) Py-Py-Im-Py, (**D**) Im-Py-Im-Py and (**E**) Im-Im-Im-Im.

To obtain insight into the differences among the observed *K*_D _values, we calculated the model structures of five 4-ring Py–Im polyamides, PyPyPyPy, ImPyPyPy, PyPyImPy, ImPyImPy and ImImImIm by DFT, as described in the ‘Material and Methods’ section. Py–Im polyamide **1** contains two PyPyPyPy, **2** contains ImPyPyPy and PyPyPyPy, **3** contains ImPyPyPy and PyPyImPy, **4** contains ImPyImPy and PyPyImPy and **5** contains two ImPyImPy. For comparison, the structure of ImImImIm was also calculated. The optimized structures and selected torsion angle of four-ring Py–Im are shown in Figure 4.

It was found that PyPyPyPy has a helical structure, and the dihedral angles of the Py carbonyl of the amide N1–C2–CO–O are ∼17°, with a slightly smaller dihedral angle at the C-terminal Py. This large dihedral angle of the Py residue is derived from a large steric hindrance between the H of C-3 in Py and the H of the contiguous amide. In clear contrast, the corresponding dihedral angles of the Im residue in the four-ring Py–Im tetramers are small (<1–2°) because of the lack of steric hindrance, and the planarity of the molecules is increased. As a result, ImImImIm becomes almost planar (Figure 4). Because of the double bond between C2 and N3 in the Im, a lone electron pair of N3 face a H of the contiguous amide, and a hydrogen bond may form between N3 in the Im and H in the contiguous amide, resulting in the shorter distance of C–N in the amide and between two Ns in the contiguous amides and the more acute angle of the two ring-to-amide bonds, compared with Py, and the coplanarity of the Im ring and the contiguous amide (Supplementary Figure S3). The angles of the two ring-to-amide bonds in each Py and Im ring were ∼147° and 138°, respectively (Supplementary Figure S3), which is consistent with a previous result (25).

We also determined the N-to-N distances at both ends of each of the four-ring Py–Im polyamides. The N-to-N distances for PyPyPyPy, ImPyPyPy, PyPyImPy, ImPyImPy and ImImImIm were 15.59, 14.83, 13.72, 12.91 and 10.64 Å, respectively (Figure 4). We also calculated the four-ring structures by *ab initio* quantum chemical calculation. The calculated structures were almost the same as those derived by DFT (Supplementary Figure S4). The calculated structural data suggest that **1** is the least curved Py–Im polyamide, and **5** is the most curved Py–Im polyamide among **1–5**.

### Implication of binding of hairpin Py–Im polyamide to DNA minor groove

It has been pointed out by the Dervan group that the twisted shape of Py–Im polyamides fits well into the minor groove of DNA. In a DNA-cyclic polyamide complex, relatively large torsion angles were observed in Im residues and Py residues (26,27). These results clearly indicate that a large conformational change is necessary for Im residues on DNA binding. The fact that the angles of the two ring-to-amide bonds in each Py and Im ring were ∼147° and 138°, respectively, which indicates that Py has less curvature, and Im has too much curvature. This also suggests that the conformational change of Im residues could lead to a match to the regular B-form DNA, and **5** requires more energy than the other four Py–Im polyamide **1–4** to change the structure for binding to the target DNA, resulting in the slowest association rate of **5** with the target DNA among **1–5**.

Dervan and coworkers also reported other Py–Im polyamides that recognize the GC-rich sequences 5′-GGGG-3′ and 5′-GGCC-3′ (24,28). In the case of the Py–Im polyamide that recognizes 5′-GGGG-3′, the association equilibrium constant (*K*_A_) is 2.8 × 10^7 ^M^−^^1^ (The *K*_D_ is 3.6 × 10^−^^8^ M) (24). The *K*_A_ of Py–Im polyamides that recognize 5′-GGGW-3′, 5′-GGWW-3′ and 5′-GWWW-3′ have been determined as 3.7 × 10^8^, 5.0 × 10^8^ and 3.5 × 10^9 ^M^−^^1^, respectively (28–31). Among these four Py–Im polyamides, the association equilibrium constant decreased as the number of Im in the Py–Im polyamides increased. However, the *K*_A_ of the Py–Im polyamide that recognizes 5′-GGCC-3′ was 9.7 × 10^9 ^M^−^^1^ (24), which is higher than that of the Py–Im polyamide that recognizes 5′-GWWW-3′. The *K*_A_ of the Py–Im polyamide that recognizes 5′-GGCW-3′ was determined to be 4.0 × 10^8 ^M^−^^1^ (32), which is similar to that of 5′-GGGW-3′. The Py–Im polyamide that recognizes 5′-GGCC-3′ may be an exceptional Py–Im polyamide, like ImPyPyPy-γ-ImPyPyPy-β-Dp that recognizes 5′-GWWC-3′ as described previously. However, the association equilibrium constant of the other three Py–Im polyamides decreased as the number of Im in the Py–Im polyamides increased. Our calculated structural data indicate that ImPyPyPy is less curved compared with PyPyImPy (Figure 4B and C). These results suggest that not only the number of Im, but also the position of Im influences the Py–Im polyamide structure and the association rate of the hairpin eight-ring Py–Im polyamide to the target DNA.

As reported previously, replacement of Py with an aliphatic β-alanine can increase binding affinity and provide flexibility in the polyamide structures, and the binding affinity of Im-β-ImPy-γ-Im-β-ImPy-β-Dp that recognizes 5′-GCGC-3′ was 100-fold over that of ImPyImPy-γ-ImPyImPy-β-Dp (3). Measurement of *K*_D_, *k*_a_ and *k*_d_ values of Py–Im polyamides containing Py/β and/or Im/β pairs is important for the next step of Py–Im polyamide design. We have also replaced two Py in **5** by β-alanine, resulting in construction of β-β-Im-β-ImPy-γ-Im-β-ImPy-β-Dp, and we measured the *K*_D_, *k*_a_ and *k*_d_ of β-β-Im-β-ImPy-γ-Im-β-ImPy-β-Dp. Interestingly, the *k*_a_ and *k*_d_ of β-β-Im-β-ImPy-γ-Im-β-ImPy-β-Dp were improved by ∼10-fold, compared with those of **5** for ODN5 or ODN10 (Y.-W. Han *et al.*, unpublished data). Further analysis of Py–Im polyamides containing Py/β and/or Im/β pairs is now in progress.

## CONCLUSION

In this study, using SPR assays, we measured the *K*_D_, *k*_a_ and *k*_d_ of Py–Im polyamides **1–5** to characterize Py and Im in hairpin Py–Im polyamides in more detail. Because *k*_a_ and *k*_d_ of some transcription factors have been determined and were contingent on the respective transcriptional factors, the measurement of *k*_a_ and *k*_d_ of Py–Im polyamides is also crucial for the design of a Py–Im polyamide as a synthetic DNA-binding module of a transcription factor. SPR data demonstrated that the *k*_d_ values of **1–5** were between 0.0039 and 0.014 s^−^^1^. The *k*_a_ values of the Py–Im polyamides decreased as the number of Im in the Py–Im polyamides increased. DFT calculations suggest that an increase in planarity, induced by the incorporation of Im, reduced the association rate of Py–Im polyamides. These data indicate that the number and the position of Im in Py–Im polyamides influence the *k*_a_ but not the *k*_d_ of the Py–Im polyamides; thus, enabling us to estimate the DNA binding kinetics of Py–Im polyamides.

We synthesized Py–Im polyamide **1**–**5** which contained two β-alanines at the N-terminal in this study, and SPR data also demonstrated that the β-Dp linker at the C-terminal of **1–5** had a slight steric preference for A•T or T•A relative to G•C or C•G.

## SUPPLEMENTARY DATA

Supplementary Data are available at NAR Online: Supplementary Table 1 and Supplementary Figures 1–4.

## FUNDING

Grant-in-Aid for Young Scientists (B) from the Ministry of Education, Culture, Sports, Science and Technology [23770204 to Y.-W.H.]; iCeMS Exploratory Grants for Junior Investigators (to Y.-W.H.); Funding Program for Next Generation World-Leading Researchers [LS072 to Y.H.]; Core Research for Evoloutional Science and Technology (CREST) of Japan Science and Technology (to H.S.) and World Premier International Research Center Initiative (WPI), MEXT, Japan (to Y.-W.H., H.Y., Y.H. and H.S.). Funding for open access charge: Grants-in-aid from the Ministry of Education, Culture, Sports, Science and Technology of Japan.

*Conflict of interest statement*. None declared.

## Supplementary Material

Supplementary Data
