# Supplementary material for: Binding of hairpin pyrrole and imidazole polyamides to DNA: relationship between torsion angle and association rate constants
Source: Nucleic Acids Res. 2012 Oct 5;40(22):11510–7. doi: 10.1093/nar/gks897 (PMC3526260; doi:10.1093/nar/gks897)
Supplement: Supplementary Data [file supp_40_22_11510__index.html]

Binding of hairpin pyrrole and imidazole polyamides to DNA: relationship between torsion angle and association rate constants — Binding of hairpin pyrrole and imidazole polyamides to DNA: relationship between torsion angle and association rate constants — Supplementary Data 

# Binding of hairpin pyrrole and imidazole polyamides to DNA: relationship between torsion angle and association rate constants

## Supplementary Data

files

**Files in this Data Supplement:**

- Supplementary Data - pdf file
