# Supplementary material for: Binding of hairpin pyrrole and imidazole polyamides to DNA: relationship between torsion angle and association rate constants
Source: Nucleic Acids Res. 2012 Oct 5;40(22):11510–7. doi: 10.1093/nar/gks897 (PMC3526260; doi:10.1093/nar/gks897)
Supplement: Supplementary Data [file supp_gks897_nar-01297-f-2012-File006.pdf]

SUPPLEMENTARY DATA

Binding of Hairpin Pyrrole and Imidazole Polyamides to DNA: Relationship between Torsion Angle and Association Rate Constants

Yong-Woon Han, Tomoko Matsumoto, Hiroaki Yokota, Gengo Kashiwazaki, Hironobu Morinaga, Kaori Hashiya, Toshikazu Bando, Yoshie Harada and Hiroshi Sugiyama

Supplementary information Contents

Table S1. Binding affinity of **1–5**

Figure S1. Sequences of 5'-biotinylated hairpin DNAs (ODN6–10).

Figure S2. SPR sensorgrams for the interaction of Py-Im polyamides with hairpin DNAs immobilized on a sensor chip SA.

Figure S3. Measurements of the calculated structures in Figure 4.

Figure S4. Measurements of the structures of 4-ring Py-Im polyamides derived by *ab initio* quantum chemical calculation.

Table S1. Binding affinity of **1–5**.

Py-Im polyamide[a]		K_D (10^{-9} M)	k_a (10^4 $M^{-1}s^{-1}$)	k_d (10^{-2} s^{-1})	ΔG^{0b} (kcal/mol)
**1**	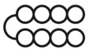	6.1 ± 0.62 (0.41)[c]	180 ± 25	1.1 ± 0.15	-11.2
**2**	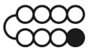	13 ± 1.0 (0.28)[d]	180 ± 31	2.3 ± 0.36	-10.8
**3**	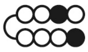	25 ± 5.8 (1.4)[e]	51 ± 24	1.3 ± 0.61	-10.4
**4**	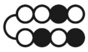	110 ± 51 (0.44)[f]	18 ± 9.3	2.0 ± 0.15	-9.5
**5**	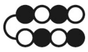	250 ± 66 (0.22)[g]	8.9 ± 4.7	2.2 ± 0.70	-9.0

[a]Closed circle and Open circle indicate Im and Py, respectively. The target DNAs of **1–5** are ODN6, 7, 8, 9 and 10, respectively.

[b]ΔG^0 values are calculated from the equation, = -RTln(1/K_D), where R is universal gas constant, 1.987 cal/mol•K; T is absolute temperature in a unit of K, here 298.15 K.

[c]The number in parentheses indicates the relative affinity to **1**/ODN1 in Table 1.

[d]The number in parentheses indicates the relative affinity to **2**/ODN2 in Table 1.

[e]The number in parentheses indicates the relative affinity to **3**/ODN3 in Table 1.

[f]The number in parentheses indicates the relative affinity to **4**/ODN4 in Table 1.

[g]The number in parentheses indicates the relative affinity to **5**/ODN5 in Table 1.

ODN6
5'-XGCC**ATATA**GGC T T
3'-CGG**TATAT**CCG T T

ODN7
5'-XGCC**ATATC**GGC T T
3'-CGG**TATAG**CCG T T

ODN8
5'-XGCC**ATAGC**GGC T T
3'-CGG**TATCG**CCG T T

ODN9
5'-XGCC**ATCGC**GGC T T
3'-CGG**TAGCG**CCG T T

ODN10
5'-XGCC**AGCGC**GGC T T
3'-CGG**TCGCG**CCG T T

Figure S1. Sequences of 5'-biotinylated hairpin DNAs (ODN6–10). X represents biotin. The binding sequences of the Py-Im polyamides are shown in bold.

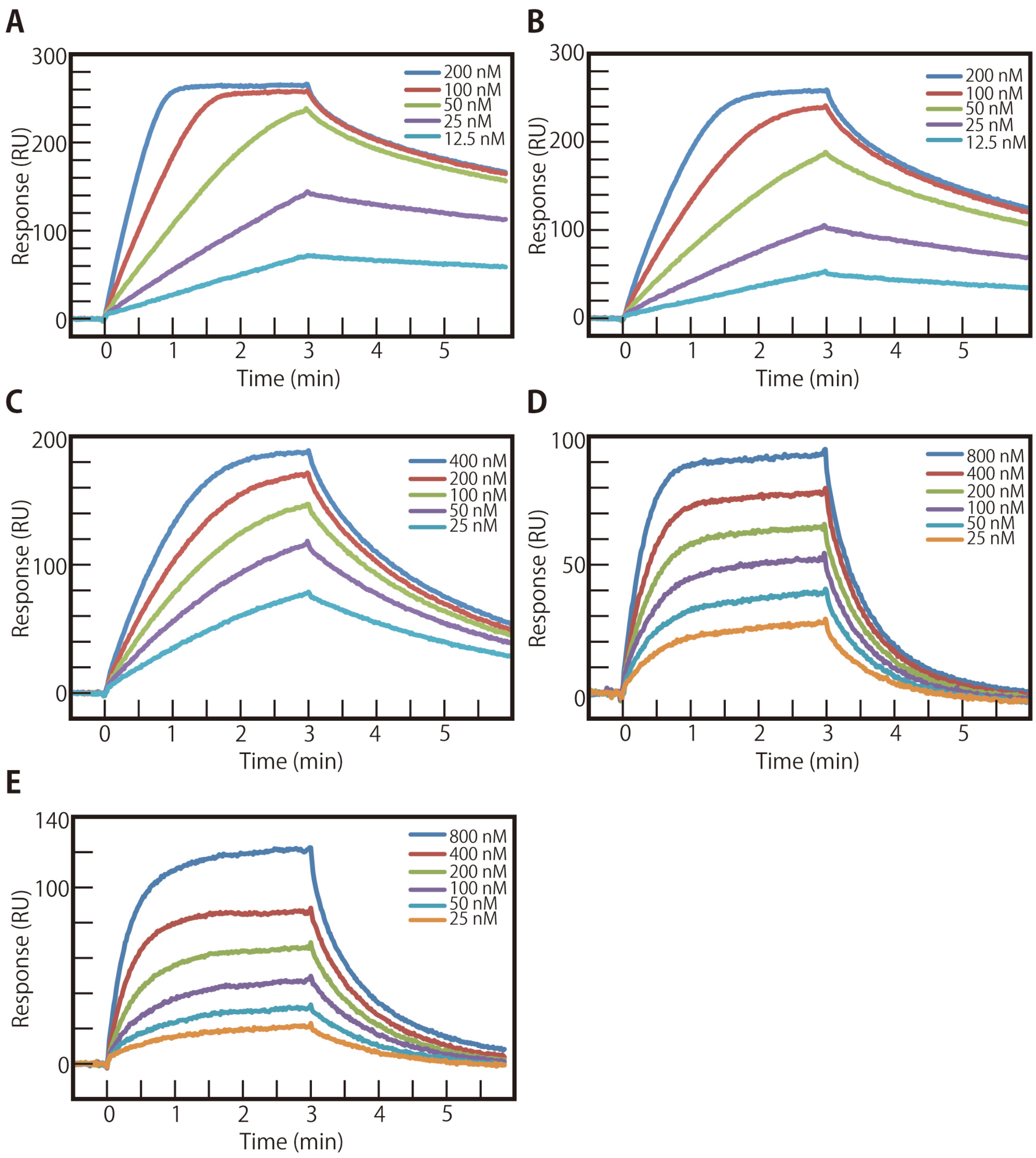

Figure S2. SPR sensorgrams for the interaction of Py-Im polyamides with hairpin DNAs immobilized on a sensor chip SA. (A) Py-Im polyamide **1** with ODN6 at a concentration range from 1.25×10^{-8} (lowest curve) to 2.0×10^{-7} M (highest curve). (B) Py-Im polyamide **2** with ODN7 at a concentration range from 1.25×10^{-8} (lowest curve) to 2.0×10^{-7} M (highest curve). (C) Py-Im polyamide **3** with ODN8 at a concentration range from 2.5×10^{-8} (lowest curve) to 4.0×10^{-7} M (highest curve). (D) Py-Im

polyamide **4** with ODN9 at a concentration range from 2.5×10^{-8} (lowest curve) to 8.0×10^{-7} M (highest curve). (E) Py-Im polyamide **5** with ODN10 at a concentration range from 2.5×10^{-8} (lowest curve) to 8.0×10^{-7} M (highest curve).

A B

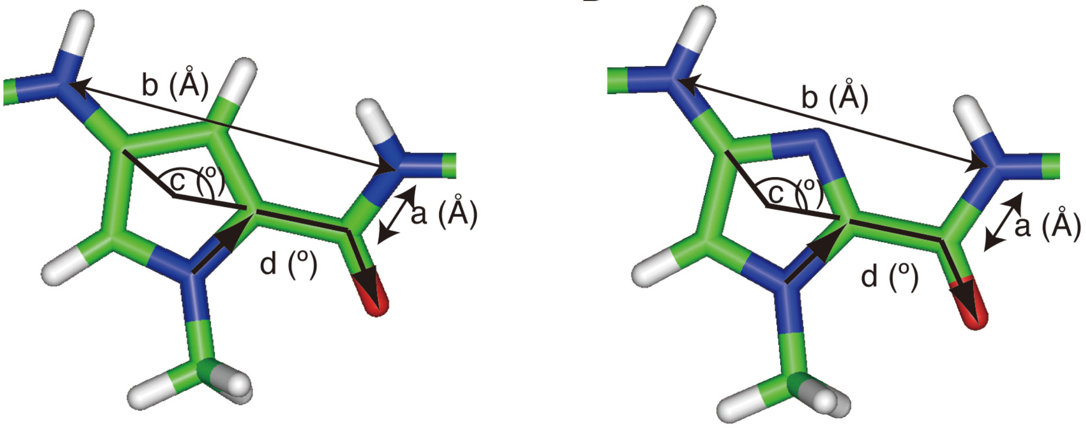

C 1 2 3 4 Py-Py-Py-Py

	**a (Å)**	**b (Å)**	**c (°)**	**d (°)**
Py1	1.38	5.45	147	16.5
Py2	1.38	5.45	147	17.9
Py3	1.38	5.45	147	17.3
Py4	1.38	5.44	147	13.3

D Im-Py-Py-Py

	**a (Å)**	**b (Å)**	**c (°)**	**d (°)**
Im1	1.36	5.09	138	0.84
Py2	1.38	5.45	148	18.1
Py3	1.38	5.45	147	17.9
Py4	1.38	5.44	147	13.1

E Py-Py-Im-Py

	**a (Å)**	**b (Å)**	**c (°)**	**d (°)**
Py1	1.38	5.45	147	16.2
Py2	1.38	5.45	147	14.4
Im3	1.36	5.10	138	0.75
Py4	1.38	5.44	147	12.3

F Im-Py-Im-Py

	**a (Å)**	**b (Å)**	**c (°)**	**d (°)**
Im1	1.36	5.09	138	0.70
Py2	1.38	5.45	148	14.1
Im3	1.36	5.10	138	0.39
Py4	1.38	5.44	147	12.7

G Im-Im-Im-Im

	**a (Å)**	**b (Å)**	**c (°)**	**d (°)**
Im1	1.37	5.10	138	0.61
Im2	1.37	5.10	138	1.20
Im3	1.36	5.09	138	2.39
Im4	1.36	5.10	138	0.21

Figure S3. Measurements of the calculated structures in Figure 4. (A) Py ring structure. a, b, c, and d indicate the C-N bond length in the contiguous amide, the distance between two Ns in the previous and the contiguous amide, the angle between the two ring-to-amide bonds in the Py ring, and the dihedral angle between the Py ring

and the contiguous amide, respectively. (B) Im ring structure. a, b, c, and d are the same as in (A). (C) The results from Py-Py-Py-Py. (D) The results from Im-Py-Py-Py. (E) The results from Py-Py-Im-Py. (F) The results from Im-Py-Im-Py. (G) The results from Im-Im-Im-Im.

A Py-Py-Py-Py (rings numbered 1 2 3 4)

	a (Å)	b (Å)	c (°)	d (°)
Py1	1.36	5.42	146	21.0
Py2	1.36	5.41	146	21.0
Py3	1.36	5.42	146	21.1
Py4	1.36	5.41	146	15.6

B Im-Py-Py-Py

	a (Å)	b (Å)	c (°)	d (°)
Im1	1.34	5.06	137	0.10
Py2	1.36	5.41	146	20.4
Py3	1.36	5.41	146	20.7
Py4	1.36	5.41	146	15.2

C Py-Py-Im-Py

	a (Å)	b (Å)	c (°)	d (°)
Py1	1.36	5.42	146	20.4
Py2	1.36	5.41	146	17.4
Im3	1.34	5.05	137	0.53
Py4	1.36	5.41	146	15.3

D Im-Py-Im-Py

	a (Å)	b (Å)	c (°)	d (°)
Im1	1.34	5.06	137	0.11
Py2	1.36	5.41	146	17.1
Im3	1.34	5.05	137	0.38
Py4	1.36	5.41	146	15.1

E Im-Im-Im-Im

	a (Å)	b (Å)	c (°)	d (°)
Im1	1.35	5.06	137	0.00
Im2	1.34	5.06	137	0.23
Im3	1.34	5.06	137	0.36
Im4	1.34	5.07	137	0.03

Figure S4. Measurements of the structures of 4-ring Py-Im polyamides derived by *ab initio* quantum chemical calculation. a, b, c, and d are the same as those in Figure S1. (A) The results for Py-Py-Py-Py. (B) The results for Im-Py-Py-Py. (C) The results for Py-Py-Im-Py. (D) The results for Im-Py-Im-Py. (E) The results for Im-Im-Im-Im.
